# Numerical study and experimental validation of the size effect of smooth and mode I cracked semi-circular bend specimens

**DOI:** 10.1038/s41598-023-34201-z

**Published:** 2023-05-10

**Authors:** Saeed Mousa, Mohammed Mutnbak, Abd-Allah M. Saba, Amr A. Abd-Elhady, Hossam El-Din M. Sallam

**Affiliations:** 1 Mechanical Engineering Department, College of Engineering, Jazan University, 706 Jazan, Saudi Arabia; 2 Civil Engineering Department, College of Engineering, Jazan University, 706 Jazan, Saudi Arabia; 3 Mechanical Design Department, Faculty of Engineering, Helwan University, Cairo, 11718 Egypt; 4 Materials Engineering Department, Zagazig University, Zagazig, 44519 Egypt

**Keywords:** Civil engineering, Mechanical engineering

## Abstract

The edge-cracked semi-circular bend (SCB) specimen subjected to three-point bending loading is used in many applications to measure the fracture behavior of quasi-brittle materials. The main objective of the present work was to study the effect of the crack length to SCB specimen radius ratio (*a/R*), span to specimen diameter ratio (*S/D*), and specimen size on its flexural and mode I crack growth behavior. The contour integral method was implemented using the 3-D finite element method to determine the mode I stress intensity factor. In addition, high-strength concrete specimens were experimentally studied to validate the numerical results. The results show that the maximum compression stress is not sensitive to the *S/D* value, while the tensile stress is very sensitive. The value of *S/D* is the main parameter controlling the crack driving force (i.e., the crack mouth opening displacement (CMOD) and the normalized stress intensity factor,* Y*_*I*_). For the same *S/D*, the SCB specimen diameter value change has a marginal effect on CMOD and *Y*_*I.*_ The specimen with *S/D* = 0.8 showed that it is the most compatible specimen with three-point bending test conditions, regardless of the SCB specimen size. A good agreement between the numerical and experimental results was achieved.

## Introduction

**Figure 1:**
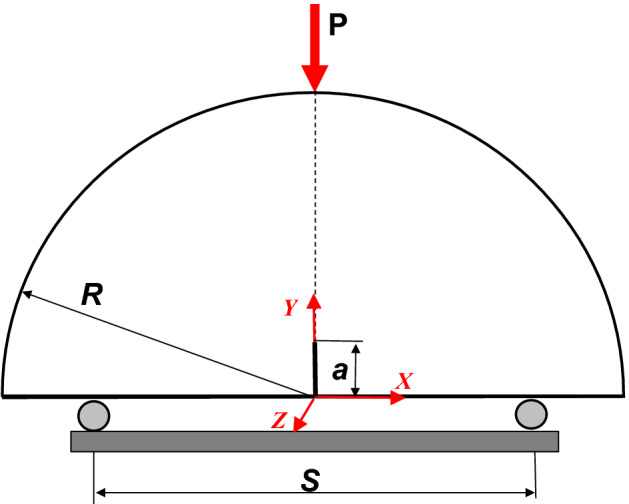
Geometry and loading conditions of SCB specimens.

The edge-cracked semi-circular bend (SCB) specimen under three-point bending loading is used to measure the material fracture behavior of rock materials, concrete, asphalt mixtures, and biomaterials^1–5^. The main advantage of using the SCB specimen is that it can easily be taken from the cores of any material^6^. Furthermore, it has a simple geometry and test procedure for calculating mixed mode I–II fracture toughness^7–9^. Arsalan et al.^10^ recently improved the SCB specimen to obtain a ductile adhesive’s mixed-mode fracture behavior with a considerable fracture process zone ahead of the crack tip. The mixed-mode SIF is a function of the crack length ratio *a/R*. Its orientation concerns the loading direction and the distance between the supports^11,12^, as shown in Fig. 1. Crack length appears to be a more significant factor than the specimen thickness on the SIF^13^. Furthermore, the SIFs become very sensitive at the large crack length to SCB specimen radius ratio (*a/R)* values^8^.

Moreover, Lim et al.^14^ studied the effect of *a/R*, span to specimen diameter ratio (*S/D*), and crack orientation on the SIF of SCB specimens under three-point bending testing. They saw that the mode II SIF becomes increasingly dominant as the support span length is reduced or when the crack angle and length are increased. They have concluded that the SIF is not as sensitive to variations in SCB specimen geometry at a short crack length. Adamson et al.^15^ used a weight function method to predict a SIF and CMOD of SCB. Furthermore, Aliha et al.^16^ used the edge-cracked SCB specimen manufactured from chopped strand glass fiber-reinforced polymer concretes under a three-point bending test to evaluate fracture toughness. In addition, they used the uncracked SCB specimen to obtain the tensile strength. The stress field around the crack tip is usually based on SIFs, crack growth, and the coefficient of the first nonsingular term^17,18^. The fracture toughness can be determined from the critical stress states or energy near the crack tip, as is required for brittle fracture initiation^19,20^. Hence, the calculation of critical stress and fracture toughness is necessary.

Many researchers^21–28^ evaluated different test specimens to measure the real fracture toughness of several brittle materials. Furthermore, many of them studied the effects of disk specimen size on fracture behavior, such as Aliha et al.^24^, who investigated the effects of the geometry and size of SCB and circular disk specimens on fracture trajectories in limestone rock under mixed-mode loading. Moreover, Abd-Elhady^22^ studied the effect of SCB specimen thickness on the mixed-mode I/II SIFs. The bending stress and the deflection of edge-cracked SCB specimens subject to three-point bending loading were considered to be the main factors responsible for propagating cracks in the specimens. Stewart et al.^6^ compared the SCB and disk-compact tension (DCT) fracture test standards^29–32^ for asphalt-aggregate mixtures. They found that the SCB tests measure a low fracture resistance with a high coefficient of variation, while the DCT test measures resistance to fracturing with a low coefficient of variation. On the contrary, Yang et al.^11^ compared three different types of three-point bend type specimens (i.e., single-edge notched beam (SENB), edge notch disk bend (ENBD), and SCB specimens) to measure the fracture toughness of asphalt mixture. The SENB specimens showed the lowest fracture toughness, while ENBD specimens showed the highest. Bažant and his colleagues^28^ stated that progress in design codes and practice for these materials had been retarded by protracted controversies about the proper mathematical form and justification of the size effect law. The dimensions of standard SCB specimens^29–31^ are 150 mm in diameter, and the ratio of the specimen thickness to its radius ratio (*B/R*) = 1/3. In addition, the *S/D* = 0.8 and *a/R* = 0.2.

The SCB specimen does not have a uniform cross-section, so the stress distribution of either a smooth or an edge-cracked SCB specimen under a three-point bending test must be fully understood to get a reliable prediction of crack growth and fracture strength. Exact solutions are not available because of the complexities of such problems. There is a lack of research studies on the effect of SCB specimen size on the mechanical and fracture behavior of cracked and uncracked SCB specimens. The main objective of the present work is to study the effects of the *a/R*, *S/D*, and SCB specimen radius on fracture behavior. Also, it considers the deflection and bending stress of edge-cracked SCB specimens subject to three-point bending loading. The three-dimensional finite element method (FEM) is employed in the present work. Furthermore, an experimental study was conducted to validate the present numerical results and to get the influence of SCB specimen size on the crack growth path, fracture force, and mode I fracture toughness (*K*_*IC*_).

## Numerical analysis

A three-dimensional finite element model was used in ABAQUS (code version 2016)^33^ to predict the mechanical and fracture behavior of SCB under a three-point bending test. In the present finite element analysis, the mechanical behavior of the SCB specimen material was assumed to be homogeneous and isotropic, showing elastic behavior. The SCB specimen of radius (*R* = *D/2*) contains an edge crack of length (*a*), as shown in Fig. 1. The specimen is carried by two bottom supports of distance *S* and is loaded by the vertical applied load equal to 5 kN. The *B/R* was kept constant while the values of *D* and *S/D* used in the present investigation are tabulated in Table 1.

**Table 1:** SCB geometry.

Symbol	Values	Description
*D*	150, 120, and 90	SCB diameter (mm)
*S/D*	0.8, 0.6, and 0.4	Distance between two support ratio
*a/R*	0.0, 0.1, 0.2, 0.3, 0.4, and 0.5	Crack length ratio

**Figure 2:**
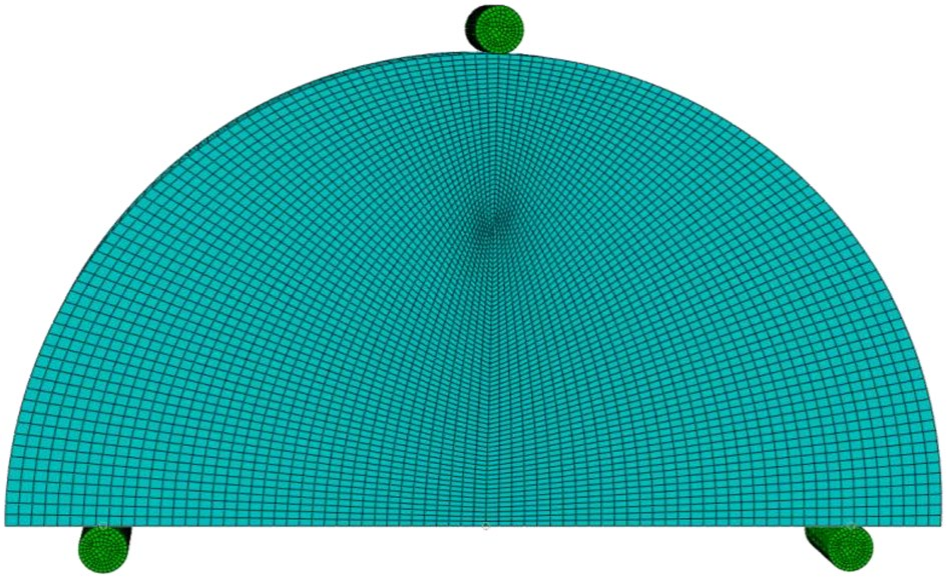
Typical 3D FEM mesh of the present model.

The SCB specimen was constructed with hexagonal structural mesh and elements of C3D8R (8-node linear brick). A mesh sensitivity test was performed to ensure accuracy in the results, as shown in Fig. 2. The contour integral method, which is a method that involves blocking the material neighboring each node along the crack line from the crack face to the opposite crack face, was used in the present simulation to extract the SIFs and *J-*integral for the SCB specimen for each crack length, *a*. SIFs are used in linear elastic fracture mechanics to distinguish the local crack-tip/crack-line stress and displacement fields. The value of *J-*integral can be calculated in ABAQUS/Standard then the SIF can be calculated through the following equation: $J= \frac{{K}^{2}}{E}$ where *E* is the modulus of elasticity. Aliha et al.^24^ and Ayatollahi et al.^25^ deduced the general formula for the mode I normalized SIF (*Y*_*I*_), which is defined as:

1 $$ K_{I} = \sigma_{ap} \,Y_{I} \sqrt {a\pi } . $$

Then

2 $$ Y_{I} = \frac{{K_{I} }}{{\sigma_{ap} \sqrt {a\pi } }}. $$

According to AASHTO TP105^29^, *Y*_*I*_ can be expressed as follows:

3 $${Y}_{I}=4.782+1.219\left(\frac{a}{R}\right)+0.063 \; exp \; \left(7.045(\frac{a}{R})\right),$$

where, *K*_*I*_ is the Mode I SIF, $\sigma_{ap} = \frac{P}{2RB}$*, B* is the specimen thickness, *P* is the applied load, *R* is the radius of the specimen, *a* is the crack length.

Furthermore, the extended FEM (XFEM) method was used to predict the crack growth path for different SCB specimen sizes. XFEM explains the crack initiation and propagation based on the maximum principal stress criterion of inelastic-brittle material. The XFEM technique depends on the phantom nodes that characterize the cracked element’s discontinuity when the fracture criterion is fulfilled. These phantom nodes are separated when the equivalent strain energy release rate exceeds the critical strain energy release rate at the crack tip. For additional details about the contour integral and extended FEMs, see Refs.^21–23^.

## Experimental works

Experimental work was performed to validate the numerical results and describe the effect of SCB specimen size on the crack growth path, fracture force, and mode I fracture toughness (*K*_*IC*_).

### Materials and mix proportions

The ordinary Portland cement (OPC) used in this research had a specific gravity of 3.15. The OPC fulfilled the Type I Portland cement requirements according to the ASTM C150^34^. Silica fume with a specific gravity of 2.3 was used. A third-generation superplasticizer, ViscoCrete-1050, was used to make homogeneous concrete. Natural sand was used as fine aggregates with a specific gravity of 2.6 in the concrete mixture. The coarse crushed granite aggregate had a specific gravity of 2.68 and a maximum size of 9.5 mm. The ratio between fine and coarse aggregate was equal to 0.37. The volume of coarse aggregate per unit volume of concrete was 0.65, as recommended by ACI 363R-10^35^. The cementitious material content was 500 kg/m^3^, and the silica fume-to-cement ratio = 0.15. The ratio of water to cementitious materials was equal to 0.33. The mixing, casting, and compaction recommendations suggested by ACI Committee 363^35^ were adopted in the present work to prepare the mix.

### Specimen preparation

**Figure 3:**
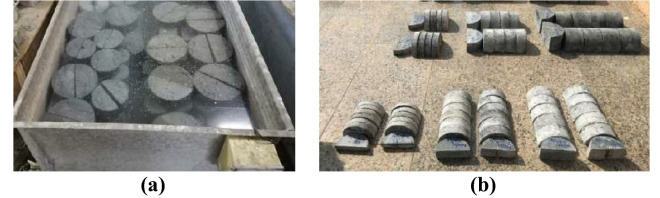
(**a**) Specimens curing in a water tank, (**b**) SCB specimens before the test.

Cubes with 100 × 100 × 100 mm dimensions were prepared to be tested under static compression. Cylinders of 100 mm in diameter and 200 mm in height were prepared to be tested under indirect tension. SCB specimens were made with 45 mm and 75 mm radii and three different B/R ratios (i.e., 0.33, 0.66, and 1) for each radius. In addition, the ratio of *a/R* was held at 0 and 0.2 for each thickness ratio. The *S/D* ratio was kept constant and equal to 0.8 in all SCB specimens. The mixed materials were placed in the molds, compacted using external vibration, leveled, and cured in water for 28 days before testing, as shown in Fig. 3.

### Test procedures

Compression and indirect tension tests were performed using a load control compression hydraulic testing machine with a 3000 kN capacity according to ACI PRC-363-10^35^. Smooth and notched SCB specimens were tested using a UTM-0108 multiplex machine with a servo motor and BC100 TFT graphics data acquisition and control system. The multiplex machine can do tests within the speed range of 0.00001 mm/min to 51 mm/min with a load capacity of 50 kN. The tests were conducted under three-point bending (3 PB) with a loading span of S, as illustrated in Fig. 1, at room temperature. Specimens were carefully placed in the fixture to ensure that the top roller was in the middle of the specimen. Then the spacing between the two bottom supports was checked to ensure the SCB specimens were similar. During the test, the load line displacement (LLD; δ) and the crack mouth opening displacement (CMOD) were measured using a linear variable differential transformer (LVDT) and clip gauge, respectively, versus the vertically applied load. In the case of smooth SCB specimens, the load and the LLD were recorded under a loading rate of 0.2 mm/min.

However, the provisions of AASHTO TP105^29^ were followed in the case of the notched SCB specimens. An initial load of 1 kN was first reached, starting from the seating load in stroke control with a rate of 0.06 mm/min. When this initial load level was reached, the system switched to CMOD control, and the load was applied such that the CMOD rate was kept constant at 0.03 mm/min for the entire test duration. The load, the CMOD, and the LLD were measured and recorded during the test.

### Ethical approval

This article does not contain any studies with human participants or animals performed by any of the authors.

## Results and discussion

### Experimental validation of the numerical results

**Figure 4:**
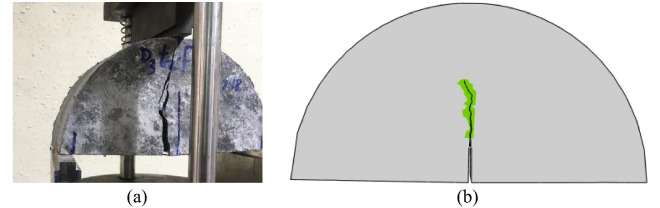
(**a**) Experimental and (**b**) numerical crack paths for cracked SCB specimen under mode I loading.

The present experimental results were used to validate the present numerical results. Figure 4 contains a visual comparison between the experimental and numerical results of the typical crack path for cracked SCB specimens under mode I loading. In all SCB specimen geometries, the crack emanated from the pre-notch root and then grew toward the applied load, as shown in the figure. There was good agreement between the numerical and experimental crack paths for cracked SCB specimens, and the SCB specimen size did not affect the crack path, as found previously by Refs.^21–23^.

As described above, the normalized mode I SIF (*Y*_*I*_) can be obtained from Eqs. (2 and 3). Furthermore, AASHTO TP105^29^ used Eq. (1) to obtain the mode I fracture toughness (i.e., the critical value of SIF, *K*_*IC*_) based on Ref.^36^. The experimental and numerical values of mode I fracture toughness of the SCB specimens are listed in Table 2. As shown in Table 2, there was good agreement between the numerical and experimental results of the mode I fracture toughness. Therefore, it can be confirmed the numerical results and depend on it.

**Table 2:** Experimental and numerical values of *K*_*IC*_ of SCB specimens.

*R* (mm)	Experimental	Numerical	Error %
	*K*_*IC*_ (MPa √mm)	*K*_*IC*_ (MPa √mm)	
45	25.12	24.54	2.3
75	28.33	27.21	3.94

**Figure 5:**
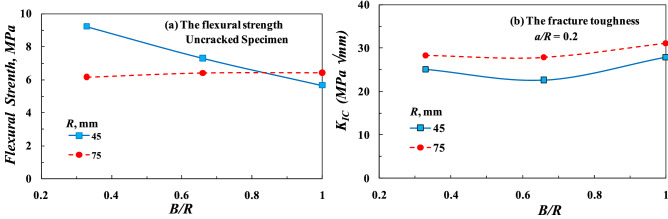
Effect of *B/R* on (**a**) the flexural strength and (**b**) the fracture toughness.

Furthermore, the effect of specimen thickness on the flexural strength and fracture toughness measured experimentally from SCB specimens is shown in Fig. 5. Specimens with R = 75 mm, which were recommended by different standards^29–31^, were marginally affected by B/R. Smooth specimens with R = 45 mm were significantly affected by *B/R*, and their flexural strength decreased markedly with increasing *B/R*. For all values of *B/R*, the values of *K*_*IC*_ measured from specimens with R = 45 mm were lower than those measured from specimens with R = 75 mm. It can be concluded that the dimensions of the recommended standard specimen^29–31^ (i.e., R = 75 mm, *S/D* = 0.8, and *B/R* = 1/3) showed reasonable results either in smooth or cracked geometry.

### Bending stress distribution of cracked and uncracked SCB specimens

**Figure 6:**
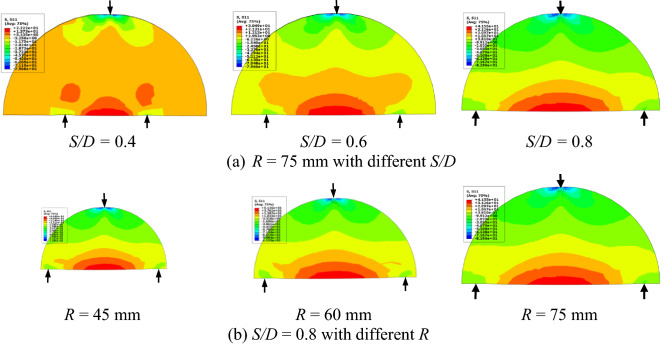
Bending stress distribution in SCB specimens with different geometries.

**Figure 7:**
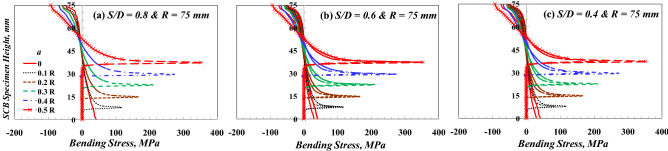
Bending stress distribution for SCB specimen with different *a* and *S/D*.

The bending stress distributions in the entire SCB specimens with different geometries (*R* = 75 mm with different *S/D* and *S/D* = 0.8 with different *R*) are presented in Fig. 6. It is clear that The patterns of the stress distributions are mainly dependent on the value of *S/D* regardless of the value of *R*. Figure 7 shows the effect of crack length on the bending stress distribution on the height of the SCB specimen subjected to three-point bending loading. In the common mechanics of materials (no defect on the body), it is well known that the maximum bending stress is located at the upper surface (*Y* = R, see the coordinate system in Fig. 1) and lower surface (*Y* = 0; see the coordinate system in Fig. 1). When *a* = 0, the peak value of this bending stress is located at the upper surface of the compression value. On the contrary, when *a* > 0, the peak value of the bending stress is transmitted to the crack tip by a higher tension value. This peak value of the maximum bending stress increases by increasing the crack length, as shown in Fig. 7.

Furthermore, the tensile bending stress at the crack tip was more than that at the lower surface at *Y* = 0 in the smooth specimen by twice to an eighth time, depending on the crack length. Moreover, the compressive stress on the upper surface is slightly affected by decreasing *S/D*. In contrast, the tension stress at the crack tip decreases.

For more clarity on the influence of *S/D* on the flexural stresses of smooth and cracked SCB specimens, Table 3 is generated. Table 3 shows that the maximum compressive stress at *Y* = R has little effect (not sensitive) by changing the value of *S/D,* but the tensile stress is sensitive to the *S/D* value. It increases by increasing the value of *S/D*. Furthermore, the coordinate of the neutral axis, which is usually in the middle of the beam height, is very sensitive to *S/D* and increases by decreasing the *S/D* value. Once again, the coordinate of the neutral axis in the standard specimen (specimen with *S/D* = 0.8) is the closest to the common value (half of the specimen height). Therefore, the standard specimen is more compatible with the bending stress conditions because it maintains the approximate symmetry in the tensioned and compressed parts of the specimen. On the contrary, the maximum tensile stress is lower than the maximum compressive stress for smooth specimens with different *S/D*. The highest ratio of the tensile to compressive stress equals 76% for the case *S/D* = 0.8.

**Table 3:** Effect of S/D on the maximum bending stresses (MPa) at *Y* = 0 and R.

*S/D*	Maximum compressive bending stress (MPa) at *Y* = R		Maximum tensile bending stress (MPa) at *Y* = 0 mm		Height of the neutral axis (*H*_*N.A.*_)/Specimen radius (*H*_*N.A.*_/*R*)	
	*a/R* = 0	*a/R* = 0.2	*a/R* = 0	*a/R* = 0.2	*a/R* = 0	*a/R* = 0.2
0.8	− 53.6	− 59.2	41	163.7	0.55	0.56
0.6	− 51.5	− 56.6	30.5	110.2	0.64	0.63
0.4	− 50.1	52.7	22.1	63.4	0.85	0.83

**Figure 8:**
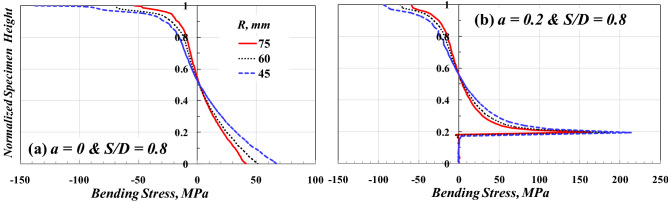
Effect of *R* on the bending stress distribution for SCB specimen.

Figure 8 shows the effects of the SCB specimen diameter on the bending stress. To improve the clarity of the figure and make it easy for comparison, the values in the vertical axis were normalized by dividing by the corresponding specimen radius (i.e., the axis ranged from 0, the maximum tensile stress in the smooth specimen, to 1, the maximum compressive stress). The diameter of the SCB specimen affects the maximum compressive stress at *Y* = R and the maximum tensile stress at *Y* = 0, in case *a* = 0. The compressive and tensile stresses increased when decreasing the value of R. A similar trend was observed for *a* = 0.2 *R*. In other words, the neutral axis of uncracked SCB specimens is located at a distance, *H*_*N.A.*_, from the lower base of 41 mm, 31 mm, and 23 mm for *R* = 75, 60, and 45 mm, respectively (i.e., *H*_*N.A.*_/*R* = 0.55, 0.51, 0.51, respectively). Moreover, the value of compression stress is higher than that of tension stress. From Figs. 7 and 8, it can be concluded that the SCB specimen with *S/D* = 0.8 is the suitable configuration in the three-point bending test regardless of the value of the SCB specimen diameter.

### Deflection of SCB specimen subject to the three-point bending test

**Figure 9:**
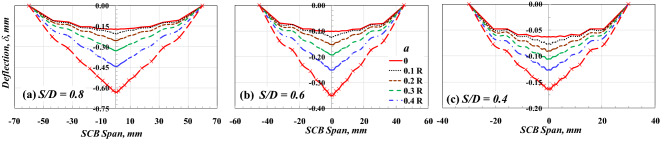
Deflection of the SCB specimen with *R* = 75 mm and different values of *a* and *S/D.*

Figure 9 illustrates the deflection of the SCB specimen under a three-point bending test for *S/D* = 0.8, 0.6, and 0.4, respectively. It is clear that the deflection increased with increasing the values of the crack length and/or the values of *S/D*, as shown in Fig. 9. Moreover, the deflection curve has a parabolic shape when the *a/R* = 0 while it has a tapered shape like a triangle shape in the case of *a/R* > 0. In the case of *S/D* = 0.8, the parabolic shape clearly appears, while in the case of *S/D* = 0.6 and 0.4, a plateau region appears in the middle of the span. This may be considered further evidence of the superiority of *S/D* = 0.8. In contrast to the conventional 3 PB specimen, the flexural stiffness (*EI*) in the SCB specimen varies along its span due to the change in its depth. In other words, the shape of the deflection curve along the span of the SCB specimen is mainly affected by the variation of the specimen depth along the beam span (i.e., the variation of the moment of inertia, *I*).

**Figure 10:**
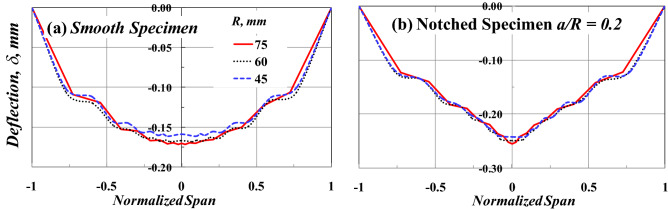
Effect of *R* on the shape of the deflection distribution along SCB specimen span with *S/D* = 0.8.

Figure 10 shows the effect of the SCB specimen radius, *R*, on the deflection of the SCB specimen under a three-point bending test. The horizontal axis in this figure is normalized by dividing its values by the corresponding specimen diameter to make a fair and clear comparison. The deflection distribution along the specimen span for both cracked and uncracked SCB specimens is marginally affected by the radius of the SCB specimen, as shown in Fig. 10a,b. It is clear from Fig. 10 that for the same *S/D* and various *R* values, the effect of the variation of the moment of inertia (*I*) on the deflection distribution along the beam span is similar. Recently, Gebhardt et al.^37^ concluded that the maximum deflection is, in most cases, not far from the mid-span of 3 PB specimens, even for biomaterial beams of the irregular cross-section.

Recall the basic concepts of mechanics of materials regarding the maximum deflection (δ_max_) of a 3-BP beam (i.e., ${\delta }_{max}=\frac{P{L}^{3}}{48 E I}$, $I=\frac{b{h}^{3}}{12}$, where *b* = beam breadth/thickness, *h* = beam height = *R* in the case of SCB specimen, *L* = beam span = *S* in the case of SCB specimen), it can be found that the deflection of the specimen made of the same material with the same thickness/breadth (*b*) is constant regardless the size of the specimen if the span to depth ratio (*S*/*R* or *L*/*h*) was constant. This concept supports the results found in Fig. 10.

**Figure 11:**
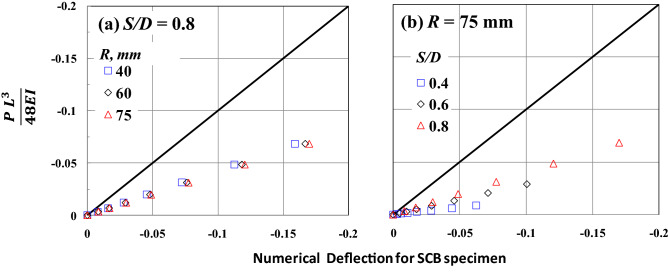
Comparison between the rectangular beam deflection and the numerical deflection of the SCB specimen is subjected to the same three-point bending loading values.

Figure 11 compares the deflection of a rectangular beam with a uniform cross-section and the deflection of the SCB specimen. The two specimens are subjected to the same values of three-point bending loading, with *a* = 0. It is worth noting that if the results are on the solid line in the figure, the deflections obtained from both specimens are the same. However, results below the solid line indicate that the deflections obtained from the SCB specimen were higher than those obtained from the conventional specimen or vice versa. For all values of *R* and *S/D*, the deflections of the SCB specimen are higher than those of the rectangular specimen subjected to the same load. For *S/D* = 0.8, the deflection of the SCB specimen has a linear relationship with the deflection of the rectangular specimen, and specimen radius, *R,* has a marginal effect on this relationship, as shown in Fig. 11a. For *R* = 75 and with changing the value of *S/D*, as shown in Fig. 11b, it can be shown that the relationship between the deflection of the SCB specimen and that of the rectangular specimen is very sensitive to the changing value of *S/D*.

### Crack tip driving force, CMOD, and mode *I* SIF of SCB specimen

**Figure 12:**
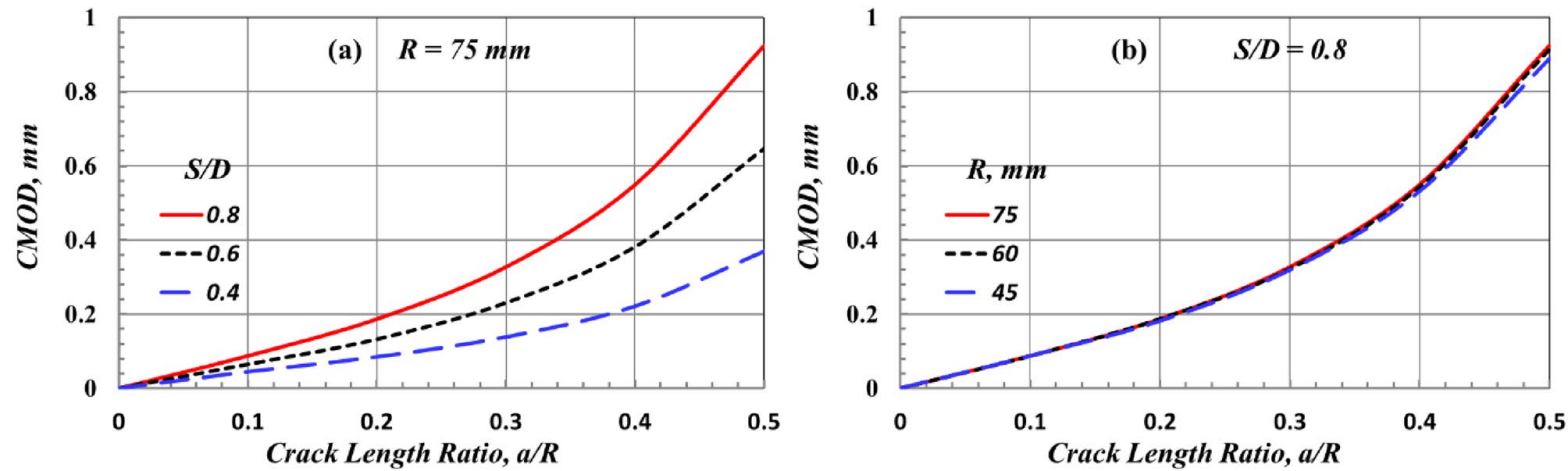
Effect of (**a**) *S/D* and (**b**) *R* on the value of CMOD of a stationary crack in SCB.

Figure 12a,b depict the effect of *S/D* and *R* on the CMOD of the crack tip of the edge-cracked SCB specimen subjected to three-point bending loading, respectively. The CMOD grows as the value of crack length *a/R* increases, regardless of the values of *S/D* or *R*. Furthermore, at the same *a/R*, the CMOD increases by increasing the value of *S/D,* as shown in Fig. 12a. Moreover, the changing value of the SCB specimen’s diameter does not affect the CMOD, as shown in Fig. 12b.

**Figure 13:**
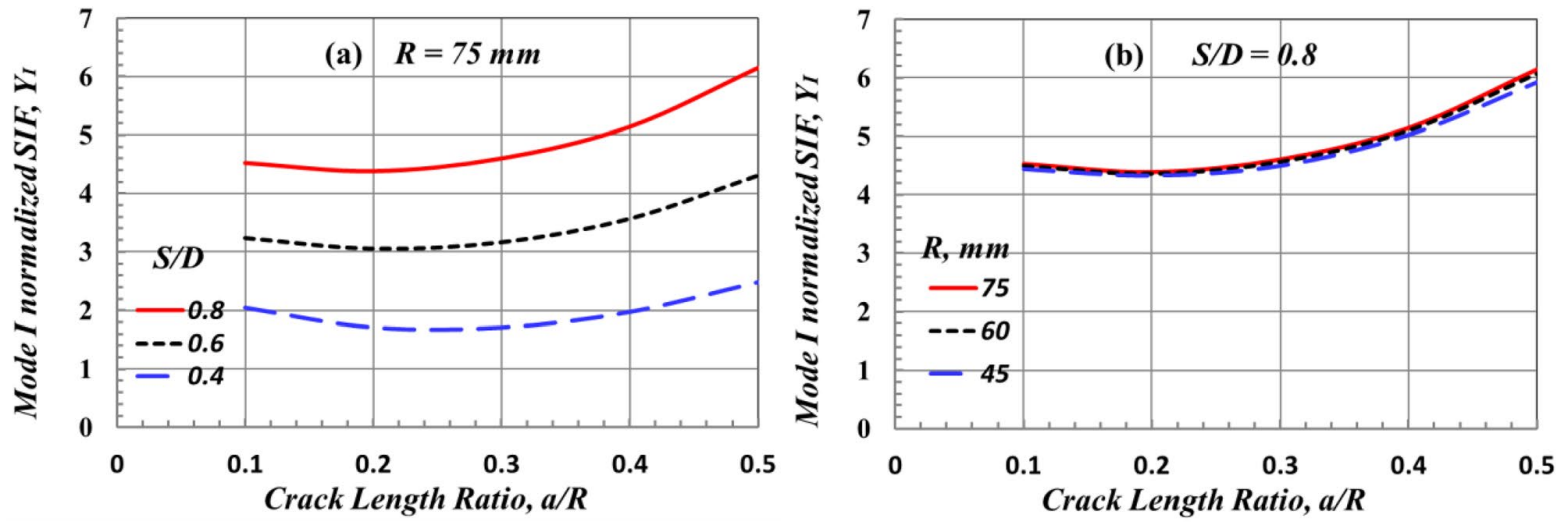
Effect of (**a**) *S/D* and (**b**) *R*on the value of mode I SIF of a stationary crack in SCB specimens due to bending load.

Figure 13a,b illustrate the effect of *S/D* and *R* on the mode I normalized SIF, *Y*_*I*_ (which was extracted by using Eqs. 1 and 2), of the edge-cracked SCB specimen subjected to a three-point bending loading, respectively. It can be seen from Fig. 13 that by increasing the crack length, the value of *Y*_*I*_ decreases to reach a minimum value, and then it increases. This finding aligns with that of Lim^8,14^. At the same *a/R*, the value of *Y*_*I*_ increases by increasing the value of *S/D*, while it is not affected by changing the value of *R*. From Figs. 12 and 13, it can be concluded that the *S/D* is the main parameter that can affect the driving force, including the CMOD and *Y*_*I*_ of the crack tip of the edge-cracked SCB specimen subjected to three-point bending loading. These driving forces, CMOD and *Y*_*I*_, also exhibited a marginal effect when the value of the SCB specimen diameter was changed.

**Figure 14:**
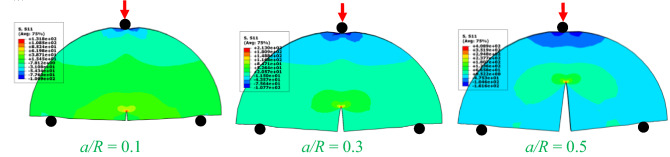
The flexural stress distribution in SCB specimens with *S/R* = 0.8 and *R* = 75 for different ratios of *a/R*.

Figure 14 shows the flexural stress contour for the notched SCB specimen for different *a/R*ratios in the case of *S/R* = 0.8 and *R* = 75 mm. Figure 14 describes the flexural stress around the crack tip. It is clear that the shape of the maximum tensile stress zone, which is presented as the process zone in brittle materials or the plastic zone in ductile materials, is a function of *a/R*. This zone expanded horizontally in the case of small *a/R* values (i.e., *a/R* = 0.1 and 0.3). However, in the case of a high *a/R* value (i.e., *a/R* = 0.5), this shape was distorted to resemble an umbrella. This may be due to a decrease in the depth of the SCB specimen from its center towards either of the two supports.

As stated earlier, the SCB specimens were suggested by AASHTO TP 105-20^29^, AASHTO TP 124-20^30^, and ASTM D8044-16^31^ for measuring the fracture toughness of asphalt mixtures [55]. Furthermore, the notched SCB specimens were used to measure the mode I fracture toughness of rock materials, concrete, asphalt mixtures, and biomaterials^1–5^. The notched SCB specimen received considerable attention emerging in 1984 to test brittle materials in particular rocks, contributing to the determination of the mode I fracture toughness because the method of rock and geometrical specimen extraction is done in a circular shape, involves simple geometry, and demands a common loading configuration. Subsequently, the SCB was adopted and improved on distinct solicitations in solid and fracture mechanics. The International Society for Rock Mechanics (ISRM) suggested using this specimen to measure the mode I fracture toughness of rock^38^.

## Conclusion

The results of the numerical analysis of the edge-cracked SCB specimen subjected to three-point bending loading support the following conclusions.

1. The compression stress at *Y* = *R* is slightly affected (not sensitive) by changing the value of *S/D,* but the tension stress is susceptible to the *S/D* value and increases by increasing the value of *S/D*. Furthermore, the highest distance, *Y*, in which the stress is in tension, is very sensitive to *S/D,* increasing by decreasing the *S/D* value. However, the opposite trend is seen with the maximum tensile stress.
2. The deflection of the SCB specimen increases as the value of crack length and *S/D* increases. Moreover, the deflection curve has a parabolic shape when the *a/R* = 0, while it has a tapered shape like a triangular shape in the case of *a/R* > 0. For *S/D* = 0.8, the deflection of the SCB specimen has a linear relationship with the deflection of the rectangular specimen, with a uniform cross-section, and specimen radius, R, has a marginal effect on this relationship. Furthermore, the deflection of the SCB specimen is higher than that of the rectangular specimen at the same load.
3. The specimen with *S/D* = 0.8 is more compatible with the three-point bending test conditions. It maintains symmetry to an approximate amount in the tension and compression part of the specimen. However, the specimen with *S/D* = 0.4 cannot be relied upon in the three-point bending test but can be used in the shear beam test.
4. The value of *S/D* is the main parameter that can affect the driving force, including the CMOD and *Y*_*I*_ of the crack tip of the edge-cracked SCB specimen subjected to three-point bending loading. These driving forces, CMOD and *Y*_*I*_, also have a marginal effect by changing the value of SCB specimen diameter at the same *S/D*.

## Data Availability

All data generated or analyzed during this study are included in this published article.

## References

[1] Sallam HEM, Abd-Elhady AA (2012). Mixed mode crack initiation and growth in notched semi-circular specimens-three dimensional finite element analysis. Asian J. Mater. Sci..

[2] Chen CS, Pan E, Amadei B (1998). Fracture mechanics analysis of cracked discs of anisotropic rock using the boundary element method. Int. J. Rock Mech. Min. Sci..

[3] Ouinas D, Bachir Bouiadjra B, Serier B, Benderdouche N, Ouinas A (2009). Numerical analysis of Brazilian bioceramic discs under diametrical compression loading. Comput. Mater. Sci..

[4] Zhao G, Yao W, Li X, Xu Y, Xia K, Chen R (2022). Influence of notch geometry on the rock fracture toughness measurement using the ISRM suggested semi-circular bend (SCB) method. Rock Mech. Rock Eng..

[5] Pijaudier-Cabot G (2022). Determination of the fracture energy of rocks from size effect tests: Application to shales and carbonate rocks. Eng. Fract. Mech..

[6] Stewart CM, Reyes JG, Garcia VM (2017). Comparison of fracture test standards for a super pavedense-graded hot mix asphalt. Eng. Fract. Mech..

[7] Khan K, Al-Shayea A (2000). Effect of specimen geometry and testing method on mixed mode I–II fracture toughness of a limestone rock from Saudi Arabia. Rock Mech. Rock Eng..

[8] Lim ILI, Johnston W, Choi SK, Boland JN (1994). Fracture testing of a soft rock with semi-circular specimens under three point bending-Part 2 Mixed mode. Int. J. Rock Mech. Min. Sci. Geomech. Abstr..

[9] Atkinson C, Smelser R, Sanche ZEJ (1982). Combined mode fracture via the cracked Brazilian disk. Int. J. Fract..

[10] Ajdani AM, Ayatollahi R, da Lucas Silva FM (2021). Mixed mode fracture analysis in a ductile adhesive using semi-circular bend (SCB) specimen. Theor. Appl. Fract. Mech..

[11] Yang D, Karimi HR, Aliha MRM (2021). Comparison of testing method effects on cracking resistance of asphalt concrete mixtures. Appl. Sci..

[12] Chang S-H, Lee C-I, Jeon S (2002). Measurement of rock fracture toughness under modes I and II and mixed-mode conditions by using disc-type specimens. Eng. Geol..

[13] Whittaker, B. N., Singh, R. N. & Sun, G. Rock fracture mechanics: Principles, design and applications. In *Developments in Geo Eng. *(Elsevier, 1992).

[14] Lim ILW, Johnston I, Choi SK (1993). Stress intensity factors for semi-circular specimens under three-point bending. Eng. Fract. Mech..

[15] Adamson RM, Dempsey JE, Mulmule SV (1996). Fracture analysis of semi-circular and semi-circular-bend geometries. Int. J. Fract..

[16] Aliha MRM, Heidari-Rarani M, Shokrieh MM, Ayatollahi MR (2012). Experimental determination of tensile strength and *K*_*Ic*_ of polymer concretes using semi-circular bend (SCB) specimens. Struct. Eng. Mech..

[17] Hutar P, Nahlik L, Knesl Z (2010). The effect of a free surface on fatigue crack behavior. Int. J. Fatigue..

[18] Mirsayar MM, Razmi A, Aliha MRM, Berto F (2018). EMTSN criterion for evaluating mixed mode I/II crack propagation in rock materials. Eng. Fract. Mech..

[19] Abd-Elhady AA, Sallam HEM, Alarifi IM, Malik TMAA, El-Bagory RA (2020). Investigation of fatigue crack propagation in steel pipeline repaired by glass fiber reinforced polymer. Compos. Struct..

[20] Mubaraki MA, Sallam HEM (2020). Reliability study on fracture and fatigue behavior of pavement materials using SCB specimen. Int. J. Pavement Eng..

[21] Mubaraki M, Abd-Elhady AA, Sallam HEM (2013). Mixed mode fracture toughness of recycled tire rubber-filled concrete for airfield rigid pavement. Int. J. Pavement Res. Technol..

[22] Abd-Elhady AA (2013). Mixed mode I/II stress intensity factors through the thickness of disc type specimens. Eng. Solid Mech. J..

[23] Mubaraki M, Abd-Elhady AA, Osman SA, Sallam HEM (2017). Mixed mode fracture behavior of concrete pavement containing RAP-3D finite element analysis. Proc. Struct. Integr. J..

[24] Aliha MRM, Ayatollahi MR, Smith DJ, Pavier MJ (2010). Geometry and size effects on fracture trajectory in a limestone rock under mixed mode loading. Eng. Fract. Mech..

[25] Ayatollahi MR, Aliha MRM, Hassani MM (2006). Mixed mode brittle fracture in PMMA—An experimental study using SCB specimens. Mater. Sci. Eng. A..

[26] Sallam HEM, Abd-Elhady AA (2013). Crack length effective stress intensity factor relation in notched semi circular specimens for different mode of mixity. Res. Appl. Mech. Eng..

[27] Mubaraki M, Osman SA, Sallam HEM (2019). Effect of RAP content on flexural behavior and fracture toughness of flexible pavement. Latin Am. J. Solids Struct..

[28] Carloni C, Cusatis G, Salviato M, Le J, Hoover CG, Bažant ZP (2019). Critical comparison of the boundary effect model with cohesive crack model and size effect law. Eng. Fract. Mech..

[29] AASHTO TP 105-20. Standard method of test for determining the fracture energy of asphalt mixtures using the semicircular bend geometry (SCB). Washington, D.C. 20001, USA 105-20 (2020).

[30] AASHTO TP 124-20. Standard method of test for determining the fracture potential of asphalt mixtures using semicircular bend geometry (SCB) at intermediate temperature. Washington, D.C. 20001, USA TP 124-20 (2020).

[31] ASTM D8044-16. Standard test method for evaluation of asphalt mixture cracking resistance using the semi-circular bend test (SCB) at intermediate temperatures. ASTM: West Conshohocken, PA, USA (2016).

[32] ASTM E7313-20. Standard Test Method for Determining Fracture Energy of Asphalt Mixtures Using the Disk-Shaped Compact Tension Geometry. ASTM: West Conshohocken, PA, USA (2020).

[33] ABAQUS. ABAQUS analysis user's guide: Technical Report ABAQUS6.14 Documentation, Simulia Corp (2016).

[34] ASTM C150-07 Standard Specification for Portland Cement. ASTM: West Conshohocken, PA, USA. www.astm.org. (2012).

[35] ACI PRC-363-10 Report on High-Strength Concrete, American Concrete Institute (ACI) (2011).

[36] Li X, Marasteanu MO (2004). Evaluation of the low temperature fracture resistance of asphalt mixtures using the semi circular bend test. J. Assoc. Asphalt Paving Technol..

[37] Gebhardt M, Steinke H, Slowik V (2023). Determination of the modulus of elasticity by bending tests of specimens with nonuniform cross section. Exp. Mech..

[38] Sofiani FM, Farahani BV, Belinha J (2022). Fracture toughness determination on an SCB specimen by meshless methods. Appl. Sci..

